# Towards automating single-particle cryo-EM data acquisition

**DOI:** 10.1107/S2052252522012039

**Published:** 2023-01-01

**Authors:** Christian Dienemann

**Affiliations:** a Max Planck Institute for Multidisciplinary Sciences, Department of Molecular Biology, Am Fassberg 11, 37077 Göttingen, Germany

**Keywords:** single-particle cryo-EM, data acquisition, automation, machine learning

## Abstract

Target selection for single-particle cryo-EM data acquisition sessions is mostly done manually by human operators, which is time consuming and leads to the inefficient use of instruments. The software toolbox *Ptolemy* [Kim *et al.* (2023). *IUCrJ*, **10**, 90–102] provides solutions for automated target selection for cryo-EM imaging.

Cryogenic electron microscopy (cryo-EM) of single particles is a powerful technique for the structural determination of biological macromolecules and significant advances in the field have been made over the last two decades (Kühlbrandt, 2014 ▸; Nogales, 2016 ▸). Improved electron detector technology (McMullan *et al.*, 2016 ▸) and data analysis algorithms (Scheres, 2012 ▸; Punjani *et al.*, 2017 ▸; Grant *et al.*, 2018 ▸; Tegunov & Cramer, 2019 ▸), as well as specialized microscope software that streamlines data acquisition (Carragher *et al.*, 2000 ▸; Mastronarde, 2005 ▸) have increased the accessibility of cryo-EM as a method for structure determination. Therefore, the number of protein and protein complex structures determined by single-particle cryo-EM is constantly increasing (see https://www.rcsb.org/stats/growth/growth-em).

For cryo-EM, a protein solution is frozen as a thin layer of vitrified ice that is embedded within a holey support film on an EM grid (Weissenberger *et al.*, 2021 ▸). Freezing of cryo-EM grids usually needs to be extensively optimized for ice layer thickness, as well as protein particle concentration and integrity, by repeating cycles of cryo-EM screening and altering sample preparation (Passmore & Russo, 2016 ▸; Noble *et al.*, 2018 ▸). Once the sample is optimized, a large number of randomly oriented particle images are acquired, classified, aligned and eventually reconstructed to a volume representing the coulomb potential density of the protein particle (Sigworth, 2016 ▸).

During cryo-EM grid screening and data acquisition, the microscope operator needs to manually pick suitable regions (squares) based on a grid overview (atlas) and select target holes with suitable ice thickness based on their appearance (Fig. 1 ▸). In many cases, ice thickness has to be chosen carefully to avoid broken or preferentially oriented particles (Noble *et al.*, 2018 ▸; D’Imprima *et al.*, 2019 ▸). Especially for the acquisition of large datasets, manual square and hole selection can be very time-consuming and less experienced operators may have difficulty targeting grid regions that yield high-quality data (Li *et al.*, 2022 ▸). Nowadays, data analysis is done ‘on-the-fly’ during acquisition (Thompson *et al.*, 2019 ▸), which gives valuable real-time information about data quality, and the microscope operator can adjust target selection based on the outcome. However, such trial-and-error strategies lead to the inefficient use of instruments that are in high demand and are expensive to maintain. Automation of the targeting of squares and holes during cryo-EM screening and data acquisition, therefore, has great potential to increase the throughput as well as the success rate of cryo-EM experiments for researchers of all experience levels.

In this issue of **IUCrJ** , Kim *et al.* (2023 ▸) present the software toolbox *Ptolemy*, which uses machine learning to automate the task of selecting target regions in single-particle cryo-EM screening and data collection. The algorithms within *Ptolemy* were pre-trained using metadata from annotated human operator microscope sessions. *Ptolemy* first addresses the automatic selection and ranking of suitable squares for data acquisition. To do so, *Ptolemy* uses a convolutional neural network [CNN, reviewed in Dhillon & Verma (2020 ▸)] classifier to predict the ‘collectability’ of squares on an atlas and can reproduce human expert operator selections on samples unknown to the neural network. *Ptolemy* then automatically finds holes on these squares using a neural network with U-Net (Ronneberger *et al.*, 2015 ▸) architecture and 2D lattice restraints for the hole positions. The U-Net not only reproduces human operator selections with high precision, but the probabilities the U-Net assigns for a hole also appear to be suitable measures for the collectability of a hole. Altogether, *Ptolemy* provides an all-in-one solution for reliable and accurate automatic targeting of squares and holes on single-particle cryo-EM grids. This is a big step towards the full automation of cryo-EM screening and data collection and is readily implemented in the microscope operation software *Leginon* [for details of the implementation, see Cheng *et al.* (2023 ▸), also published in this issue of **IUCrJ** ].

While *Ptolemy* uses specifically tailored and tuned CNN and U-Net machine-learning approaches to achieve high accuracy for recognizing and ranking squares and holes, other software have approached the problem of automatic data acquisition in slightly different ways. A conceptually similar approach was taken by *SmartScope* (Bouvette *et al.*, 2022 ▸), which utilized dedicated square and hole finders to select targets for the operator. In comparison to *Ptolemy*, the *SmartScope* square and hole recognition procedures are based on an R-CNN with ResNet50 architecture and a YOLOv5 model with CSPNet backbone for square and hole recognition, respectively. It remains to be seen which deep-learning implementation yields better performance in real-life cryo-EM imaging sessions. Notably, *SmartScope* implemented *Ptolemy* as an alternative to their own square and hole recognition algorithms (Bouvette & Viverette, 2022 ▸), so direct comparison will be possible.

A conceptually different approach is taken by *cryoRL* (Li *et al.*, 2022 ▸). Instead of attempting to generate a complete selection of suitable squares and holes prior to cryo-EM imaging, *cryoRL* treats the selection of imaging targets as a path-planning problem where the algorithm is rewarded when imaging good targets. Currently, a target is considered good when it yields a cryo-EM image with high information content, which inversely correlates with ice layer thickness. However, the thinnest ice layer possible might not be a suitable target for acquiring data of sensitive or very large protein complexes (D’Imprima *et al.*, 2019 ▸; Noble *et al.*, 2018 ▸). Instead, other results from ‘on-the-fly’ data analysis, like complex integrity, particle number per image or the orientation distribution of particle views in the 3D reconstructions, could represent suitable quality targets.

It seems likely that combining the approaches taken by *Ptolemy*, *SmartScope* and *cryoRL* will lead to very powerful automatic cryo-EM data acquisition tools. Such tools would first generate highly accurate initial collectability rankings of squares and holes, whereas the process of data collection would be guided by sample-specific ‘on-the-fly’ decision-making that is based on data analysis results.

## Figures and Tables

**Figure 1:**
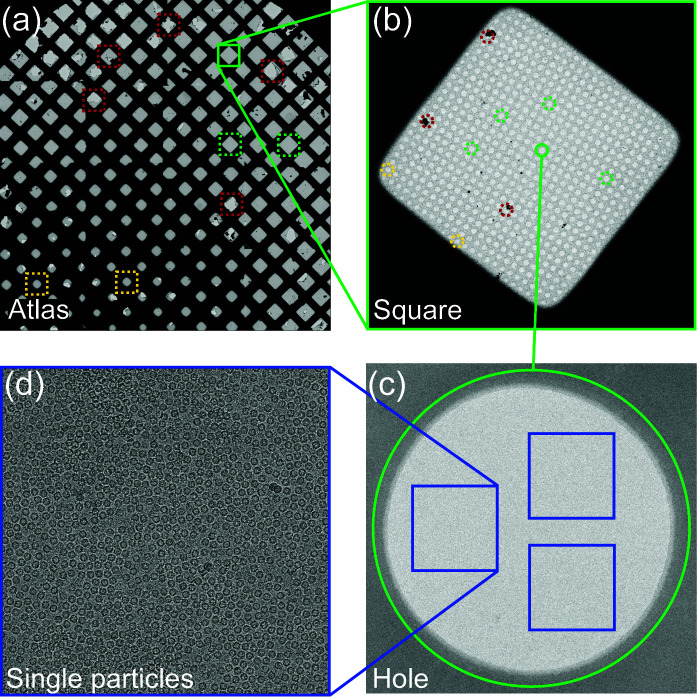
Required target selection steps during single-particle cryo-EM data acquisition. (*a*) Low magnification overview (Atlas) of a cryo-EM grid. Human operator choices for squares that are suitable for imaging are marked with green boxes. Yellow and red boxes represent operator choices of squares with thick ice layers or broken support film, respectively. (*b*) Square with examples of operator choices of holes that are suitable for cryo-EM imaging marked with green circles. Yellow and red circles mark operator choices of holes that have suboptimal ice thickness or are covered by ice contamination, respectively. (*c*) High-magnification image of a hole with a thin ice layer. Areas for cryo-EM data acquisition are marked with blue boxes. (*d*) Cryo-EM image acquired under optimal conditions and in thin ice showing single particles of good density.
